# Grafted Composite Decision Tree: Adaptive Online Fault Diagnosis with Automated Robot Measurements

**DOI:** 10.3390/s25216530

**Published:** 2025-10-23

**Authors:** Sungmin Kim, Youndo Do, Fan Zhang

**Affiliations:** George W. Woodruff School of Mechanical Engineering, Georgia Institute of Technology, Atlanta, GA 30332, USA; sungmin@gatech.edu (S.K.); doyoundo@gatech.edu (Y.D.)

**Keywords:** decision tree, adaptive fault diagnosis, measurement selection, robotic inspection, online monitoring

## Abstract

In many industrial facilities, online monitoring systems have improved the reliability of key equipment, reducing the cost of operation and maintenance over recent decades. However, it often requires additional on-site inspection of target facilities due to limited information from installed sensors. To systematically automate such processes, an adaptive online fault diagnosis framework is required, which consecutively selects variables to measure and updates its inference with additional information at each measurement step. In this paper, adaptive online fault detection models—grafted composite decision trees—are proposed for such a framework. While conventional decision trees themselves can serve two required objectives of the framework, information from monitored variables can be less utilized because decision trees do not consider if required input variables are always monitored when the models are trained. On the other hand, the proposed grafted composite decision tree models are designed to fully utilize both monitored and robot-measured variables at any stage in a given measurement sequence by grafting two types of trees together: a prior-tree trained only with observed variables and sub-trees trained with robot-measurable variables. The proposed method was validated on a cooling water system in a nuclear power plant with multiple leak scenarios, in which improved measurement selection and increase in inference confidence in each measurement step are demonstrated. The performance comparison between the proposed models and the conventional decision tree model clearly illustrates how the acquired information is fully utilized for the best inference while providing the best choice of the next variable to measure, maximizing information gain at the same time.

## 1. Introduction

### 1.1. Background

Over the past decades, online monitoring systems have consistently transformed the paradigm of operation and maintenance across a wide range of industries [1]. Before the emergence of online monitoring systems, engineers had to manually observe the facilities to support decision-making for maintaining machines’ operability. However, as sensor technologies and data management evolved, online monitoring systems quickly expanded their coverage, ensuring improved reliability and productivity of the facilities with less effort. More recently, industries are facing an even bigger transition moment driven by the brilliant advances of artificial intelligence [2]. Thanks to the enormous amount of data continuously streamed into the monitoring systems, industrial facilities are one of the ideal places where data-driven models can provide huge impacts on evaluating conditions of the facilities and have expedited studies on predictive maintenance as well.

Despite their notable contributions, online monitoring systems have their inherent limitation that they can never cover all the details of the facilities due to the finite nature of the number of sensors. Human operators are often required to visit the facilities to collect additional data for an accurate diagnosis of faults, especially the ones not considered in the design stage of the online monitoring systems. Deploying humans to facilities brings a number of issues: safety risks, inaccuracy in diagnosis, and additional costs. To circumvent those issues, robots are actively being considered as one of the promising alternative solutions for on-site inspection tasks not only by researchers but also by operators of diverse industrial facilities. With those efforts, the inspection robot market is continuously growing and expected to reach USD 9.8 billion by 2034 [3].

Although many industries are increasingly adopting robots for condition monitoring and assessment, robots tend to be operated on an independent system instead of being incorporated into the existing online monitoring system with installed sensors in most cases so far. It is because online monitoring systems are designed to provide important information to human operators, not to additional robotic systems. This leaves integrating information from different sources into a single conclusion to human experts who have access to both systems. However, robots are agents that can interact more systematically with online monitoring systems and are expected to provide greater value when they are seamlessly integrated with online monitoring systems. Such well-blended robot operation systems would minimize safety risks and costs in fault diagnosis while providing consistent and accurate inferences promptly whenever required.

**Figure 1:**
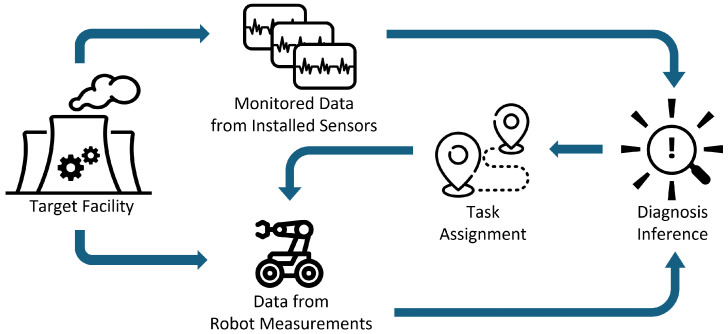
Online monitoring system with automated robot inspection.

In this paper, a machine learning (ML)-based fault diagnosis framework, which integrates robot measurements with an existing online monitoring system, is explored (Figure 1). In this framework, data acquisition tasks for robots are determined based on the information from the monitoring systems with installed sensors first. Once robots acquire additional data according to the given tasks, the diagnosis ML model updates its inference result with the new data in addition to one it was relying on. If the ML model determines the inference can be further improved by additional data, it again issues an additional order to the robots. This recursive process continues until the model concludes no more inference improvement is needed or can be achieved.

The major two required functions of the diagnosis ML model in this framework are as follows:

1. Providing a sequence of measurement requests to inspection robots based on the best information the model has, and
2. Predicting the status of target facilities with all acquired information across all stages of robot measurement processes.

To satisfy these purposes, grafted composite decision trees (GCDTs) are proposed for the diagnosis model used in the framework in this study. Because decision trees (DTs) compare variables one by one according to the feature importance of the given training set, and incrementally reduce uncertainties in their prediction, the original DT models themselves are greatly suited to delivering those two objectives: measurement selections, and inference with limited features. While leveraging those inherent advantages of DTs, the GCDT models are further improved to better utilize prior information provided by installed sensors in selecting variables to measure. Two GCDT models, layered decision tree (LDT) and adaptive decision tree (ADT), are presented in this paper.

This paper starts with reviewing the related studies regarding measurement selection problems and inference with partially observable environments. Detailed descriptions of the model architecture, and training and inference processes of the proposed models are followed. The next section describes how the models were validated on a case study with a digital twin of a nuclear power plant facility in diagnosing leaks from pipes where a number of sensors are installed providing monitored variable data. The Result section compares the performance of the proposed models with a conventional DT model in terms of accuracy and uncertainty over measurement steps. It also demonstrates the superiority of the proposed models in a physical space that incorporates the time costs of the robot traveling between measurement locations. Finally, this paper concludes with a summary of the experiments, implications of the proposed methods, and future works that could further improve both the performance and applications of the presented models.

### 1.2. Related Studies

The measurement selection problem has been studied since the early ages of ML studies [4]. However, most relevant studies are performed under the terminology “feature selection” or ”variable selection”. Feature selection is a process of finding a subset of the most effective features among the originally given features. It selects the input variables to ML models to reduce variance and achieve better performances, including improved accuracy, reduced overfitting, and improved interpretability [5]. Not only are more and more studies being reported on this realm, but also massive review papers are being published consistently [6,7,8,9,10,11].

Most of the review papers categorize feature selection methods into three: filter methods, wrapper methods, and embodied methods. Filter methods use data characteristics to evaluate features without the use of classification algorithms [12]. Relief and its variants, which work based on the distance between instances on each feature [13], and information gain, which focuses on the entropy of target values with respect to each feature [14], are the most representative techniques in this category. Wrapper methods choose the feature subset through the evaluation processes of a predetermined learning algorithm [15]. Depending on the order of evaluation of the subsets, this category is further classified into forward selection, backward elimination, and bidirectional selection. Embedded methods perform feature selection during the modeling algorithm’s execution [8]. The most representative example is learning algorithms of classification and regression tree (CART) [16,17].

However, the measurement selection problem addressed in this research has a number of different aspects compared to feature selection. First, measurement selection is the process used only in inference, not in the training process, while feature selection serves the datasets for both the training and evaluation of the models. This research assumes that the models are trained on all accessible features, but only limited features are available when the model is deployed. Second, the measurement selection is designed to choose features depending of the values on the previous observation. This research explores techniques to modify well-studied feature selection methods to meet the purpose of the proposed robot operation framework, focusing on these two differences.

Inference with limited input data is a relatively well-studied topic as well. Missing data often refers to parts of input features that are missing in each sample. Missing data problems are addressed mainly by how to impute those missing features from other sources [18]. Partially observable Markov decision processes (POMDP) are one of the well-studied decision-making systems with partially observable setups [19,20,21]. POMDP is based on the Markov decision process (MDP), which was introduced in the 1950s [22], and now well recognized as the canonical expression for environment setup in reinforcement learning, especially when the state is not fully observable [23]. POMDP is defined by several elements, including state, action and observation, which can be given by the fault mode of the target facilities, choices on variables to measure, and measured data, respectively, to express diagnosis with a robot as an agent in the given environment. However, reinforcement learning, the most practical solution to POMDP to date, still struggles with several significant challenges such as sample inefficiency, the curse of dimensionality, instability of training, and so on [24]. Because of those limitations, this research utilizes DT models instead, which are much simpler and computationally less demanding but still powerful and explainable, to solve inference with partially observed data.

## 2. Methods

**Figure 2:**
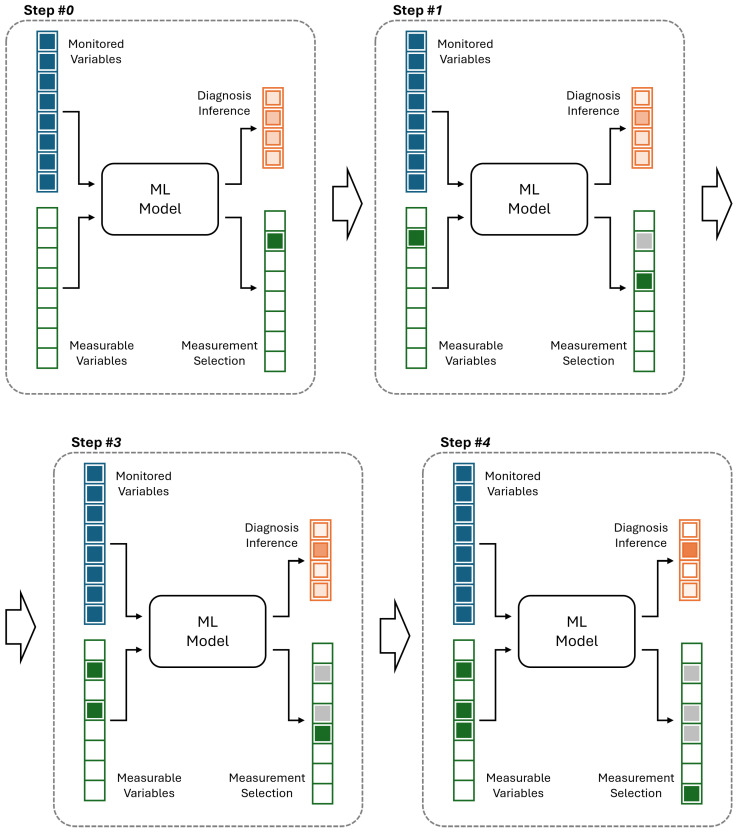
Example of consecutive measurement selection with an ML model.

Figure 2 illustrates how the ML model operates within the framework proposed in Figure 1. The model receives both monitored and measurable variables as inputs and produces two outputs: diagnostic inference and measurement selection for the robot. Each cluster of boxes represents a vector, with individual boxes denoting its elements. At measurement step #0, i.e., before the robot begins measurements, all monitored variables are available to the model, shown as filled boxes, while none of the measurable variables are yet available, shown as empty boxes. Using these inputs, the ML model generates diagnostic inference in terms of class probabilities, represented by color thicknesses, and simultaneously recommends which measurable variable should be collected next to maximize information gain, represented by a filled box among other empty boxes. At step #1, the model incorporates both the monitored variables and the newly measured variable from the previous step, yielding refined diagnostic inference and a new recommendation for the next measurement. Note that the number of candidate variables to be measured is reduced by one compared to the previous step, as one variable has already been measured and is represented by a gray box. This iterative process continues through subsequent steps. At each step, one additional measurable variable is incorporated, and the diagnostic inference becomes increasingly confident, as reflected by the progressively sharper distinctions in color thickness among class probabilities in the figure. The cycle repeats until the inference is sufficiently confident to support a final decision, which is not restricted to step #4 as it is in this example.

DT was chosen as the base model to deliver the required functions illustrated in Figure 2. DT is one of the most well-known ML models for its simplicity and explainability. It also serves as the base model in many powerful ensemble models—such as random forest and boosting models [25,26,27]. A DT is made up of consecutive splitting nodes. In classification tasks, each node makes a decision based on a single feature among many of the given data, which maximizes information gain; thus, the whole model can achieve the lowest impurity under given regularization constraints. In the training process of DT, all possible splits by a single feature of a given training set are compared at each node, and the split that can decrease total impurity most is selected for that node [16]. This inherently results in providing a sequence of features on the nodes used in its inference process. By comparing a feature value with the threshold in each node, the next feature to compare for its best prediction at that step is decided. This characteristic of DTs best fits both objectives of the diagnostic ML model for automated robot inspection framework stated in the Introduction.

Meanwhile, many industrial facilities have monitoring systems with a number of installed sensors, which continuously provide insightful information on the status of the facilities even before robot measurement starts. Although monitored variables are always available for diagnostic models, conventional DTs fail to prioritize those in its prediction because their architectures do not consider which variables are being monitored.

**Figure 3:**
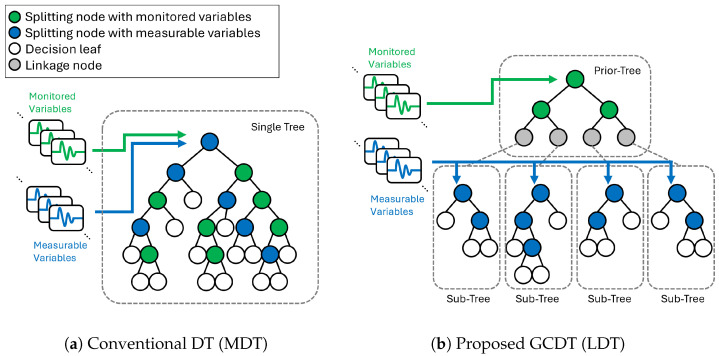
Comparison between conventional DT (MDT) and proposed GCDT (LDT).

Figure 3a illustrates how a conventional DT model is built when both monitored variables and measurable variables are provided together. “Monitored variables” refers to the ones being streamed by installed sensors, and ”measurable variables” are the ones that do not have installed sensors but can be acquired as a robot performs measurements on them. The green nodes are the ones that split the given data based on a monitored variable, and the blue nodes are the ones that split the data based on a robot-measurable variable.

When deployed, the model starts its prediction from the root node at the top of the tree. Each node in the tree stores its predicted label and uncertainty of the prediction, which are determined by the statistics of the training dataset that reached the node during training. Each node also stores which child nodes to proceed according to the split, which is also determined during training. In this manner, DT requires variables one by one as it moves towards deeper nodes. As inference gets to deeper nodes in the tree, uncertainty in prediction is reduced, and more accurate predictions can be achieved. This consecutive inference update continues until the inference reaches the leaf nodes, which do not have any split to child nodes.

As illustrated in Figure 3a, because DTs do not discriminate between monitored variables and measurable variables, nodes at shallow depths in the DT can require measurable variables at early stages of their inference. This results in less utilizing information from monitored variables in the early stage of robot measurements. For example, when the root split feature is a measurable variable, the model cannot make any decision until the first measurement is completed, even though all monitored variables are available for it. Due to this inefficiency and uncontrolled selection of feature types of the nodes, this conventional DT model is called a mixed decision tree (MDT) in this study.

In order to overcome the limitation of MDT models, this research proposes a new architecture of tree model, the grafted composite decision tree (GCDT). The key idea of the proposed GCDT models is having two completely separated types of trees: a “prior-tree” only with monitored variables and “sub-trees” with measurable variables in addition to monitored variables. By grafting these heterogeneous trees together, the model can not only maximally utilize prior information from installed sensors first in diagnosing the target facility even before the robot measurement starts, but also provide the best measurement selection for the robot in every single step depending on the known variables up to that step.

In this paper, two types of GCDT models are presented: a layered decision tree (LDT) and an adaptive decision tree (ADT). LDT, a two-layer tree model, feeds monitored variables and measurable variables into two different layers of trees, as illustrated in Figure 3b. ADT is a multi-layer tree model that expands and generalizes the key concepts of LDT to maximize information from known variables at each measurement step, which will be described in details in the later section.

### 2.1. Layered Decision Tree (LDT)

LDT is a model composed of two layers of trees, a prior-tree, and one layer of sub-trees as shown in Figure 3b. The prior-tree is trained only with the monitored variables first. On each leaf node of the prior-tree, a new sub-tree is grafted, which is trained only with measurable variables. This enables utilizing monitored variables fully before the model requires additional measurements. The detailed training process of LDT is described in Algorithm 1 with the following annotations:

- $x_{\mathrm{train}}\in R^{I_{\mathrm{train}}\times J}$: training data samples.
- $x_{\mathrm{test}}\in R^{I_{\mathrm{test}}\times J}$: test data samples.
- $y_{\mathrm{train}}\in R^{I_{\mathrm{train}}}$: training label samples.
- $y_{\mathrm{test}}\in R^{I_{\mathrm{test}}}$: test label samples.
- $y_{\mathrm{pred}}\in {\left\{y\in N_{0}|y<C\right\}}^{I}$: predicted classes.
- I∈N: Number samples.
- J∈N: Number all features.
- K∈N: Number of trees.
- C∈N: Number of classes.
- $N_{k}\in N$: Number of nodes in tree $T_{k}$.
- $L_{k}\subset \left\{n\in N_{0}|n<N_{k}\right\}$: Set of node indices of leaves in tree $T_{k}$.
- i∈{0,1,…,I−1}: sample index.
- j∈{0,1,…,J−1}: feature index.
- k∈{0,1,…,K−1}: tree index, k=0 is the index for the prior-tree.
- $n\in \{0,1,\ldots ,N_{k}-1\}$: node index, n=0 is the index for the root node.
- $V_{M}$: Set of monitored feature indices.
- $V_{R}$: Set of measurable feature indices.
- $V=V_{M}\cup V_{R}$: Set of all feature indices, (|V|=J).
- $T_{k}$: *k*th tree in the model.
- $T_{k,n}$: *n*th node in $T_{k}$.
- *X*: Set of all sample indices.
- $X_{k}$: Set of sample indices given to $T_{k}$, ($X_{0}=X$).
- $X_{k,n}$: Set of sample indices given to node *n* in tree $T_{k}$, ($X_{k,0}=X_{k}$).
- $y_{k,n}$: Nodal class decided by majority label of $X_{k,n}$ during training.

**Algorithm 1** LDT training process  1:k←0  2:Define a prior-tree, $T_{0}$  3:Train $T_{0}$ with $x_{\mathrm{train}}\left(X,V_{M}\right)$  4:**for** $n\in L_{0}$ **do**  5:      k←k+1  6:      Define a sub tree $T_{k}$  7:      $X_{k}\leftarrow X_{0,n}$  8:      Train $T_{k}$ with $x_{\mathrm{train}}\left(X_{k},V_{R}\right)$  9:      $T_{0,n}\leftarrow T_{k,0}$ (Graft $T_{k}$ to the leaf of $T_{0}$)10:**end for**

Inference with the trained LDT is quite similar to that of conventional DTs. Once samples are fed to the root of the prior-tree, the model consecutively determines inference paths through the nodes until reaching a leaf. Unlike MDT, the prior-tree in LDT would not be interrupted in the middle because all features required by the nodes on the inference path are all monitored variables and always available. However, when the inference reaches a leaf where a sub-tree is grafted, it starts to require a new measurement, which is decided by each node in the sub-tree. Once required data is acquired by robots, the inference can move on to one of the child nodes in the next depth and make a new prediction on the status of the target facilities. It is also worth noting that sub-trees take samples only from the corresponding leaf nodes of the prior-tree. It means that only a single sub-tree is activated when a single sample inference is performed, as only a single node per depth is used for a single sample inference with DT models. For batch inference with multiple samples for test or validation purposes, Algorithm 2 can be used for efficient computation.
**Algorithm 2** LDT testing process1:k←02:Run the prior-tree $T_{0}$, with $x_{\mathrm{test}}\left(X,V_{M}\right)$ and $y_{\mathrm{test}}\left(X\right)$3:**for** $n\in L_{0}$ **do**4:      k←k+15:      $X_{k}\leftarrow X_{0,n}$6:      Run the sub tree $T_{k}$ grafted at the leaf with $x_{\mathrm{test}}\left(X_{k},V_{R}\right)$ and $y_{\mathrm{test}}\left(X_{k}\right)$7:      $y_{\mathrm{pred}}\left(X_{k,n}\right)\leftarrow y_{k,n}$8:**end for**

### 2.2. Adaptive Decision Tree (ADT)

ADT is a model composed of multiple layers of DTs. It is designed to maximize confidence of its inference at each measurement step. The key difference between ADT and LDT is the number of layers in the model architecture. While LDT has a single layer of sub-trees and each node in the sub-trees represents a single measurement step, ADT consists of multiple layers of sub-trees, and each single sub-tree represents a single measurement step. It is designed to compensate for the limitation of the greedy algorithm used in DTs. Because greedy algorithms choose the best option at each step, they can result in sequences that are not globally optimized. ADT overcomes this issue by updating the tree model with all known variables up to the step, both monitored variables and variables measured in the preceding steps. The training process of ADT is described in Algorithm 3, with the following additional annotations:

- $x_{\mathrm{val}}\in R^{I_{\mathrm{val}}\times J}$: validation data samples.
- $y_{\mathrm{val}}\in R^{I_{\mathrm{val}}}$: validation label samples.
- m∈{0,1,…,M}: Measurement step index.
- M∈N: Maximum measurement steps.
- K∈N: Number of trees being parents to other trees.
- $K^{\prime }\in \left\{K^{\prime }\in N|K^{\prime }\geq K\right\}$: Number of trees including child trees being built.
- k∈{0,1,…,K−1}: parent tree index, k=0 is the index for the prior-tree.
- $k^{\prime }\in \left\{K,K+1,\ldots ,K^{\prime }\right\}$: index of child tree being built.
- $T^{\prime }$: Prospective tree.
- $V_{k}^{o}$: Set of known variables for parent tree $T_{k}$.
- $V_{k^{\prime }}^{o}$: Set of known variables for child tree $T_{k^{\prime }}$.
- $j_{\mathrm{sel},k^{\prime }}$: selected variable index for the child tree $T_{k^{\prime }}$.
- $E_{k}$: Set of leaf node indices in $T_{k}$, that do not have a connected child tree.

**Figure 4:**
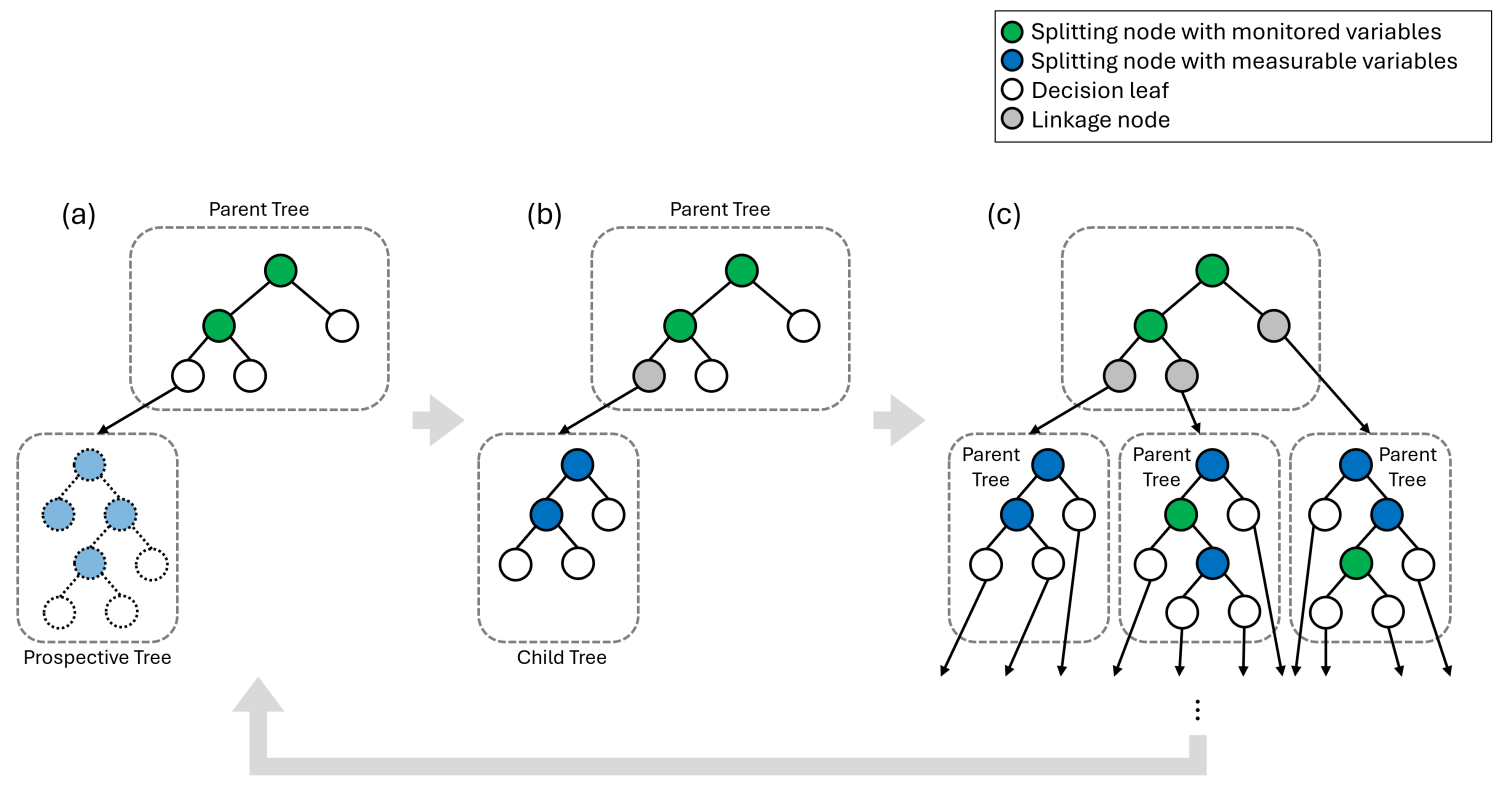
Recursive process of building ADT. (**a**) Create a prospective tree from a leaf of the parent tree with measurable variables and choose variables to measure, (**b**) Replace the prospective tree with a child tree trained with selected variables, (**c**) Complete building child trees on all leaves, and repeat building next generation trees.

Figure 4 is a graphic illustration of the training process of ADT described in Algorithm 3. First, a prior-tree ($T_{0}$) is trained with monitored features ($V_{0}^{o}=V_{M}$). In step (a) of Figure 4, with the prior-tree as the parent tree, the leaves of the parent tree ($L_{0}$) are identified. With samples reached to a leaf ($X_{0,n\in E_{0}}$), and unmeasured variables ($V\setminus V_{k}^{o}$), a prospective tree ($T^{\prime }$) is built. The purpose of the prospective tree ($T^{\prime }$) is to select the variable to measure that adds the most useful information to diagnose the target facilities among all unmeasured variables. The feature to measure which index is $j_{\mathrm{sel}}$ is chosen by its highest feature importance in the prospective tree ($T^{\prime }$).

In step (b) of Figure 4, once the selected feature is determined, the prospective tree is discarded and a new child tree ($T_{k^{\prime }}$) is built. The new child tree is trained with samples reaching to a leaf ($X_{k^{\prime }}\leftarrow X_{k,n}$), and updated known variables ($V_{k^{\prime }}^{o}\leftarrow V_{k}^{o}\cup \left\{j_{\mathrm{sel}}\right\}$). The trained child tree is grafted to the leaf of the parent tree, and the leaf of the parent tree becomes a linkage node to the child tree.
**Algorithm 3** ADT training process  1:Define a prior-tree, $T_{0}$  2:Train $T_{0}$ with $x_{\mathrm{train}}\left(X,V_{M}\right)$ and $y_{\mathrm{train}}\left(X\right)$  3:$V_{0}^{o}\leftarrow V_{M}$  4:K←1  5:$E_{0}\leftarrow L_{0}$  6:**for** m=1,2,…,M **do**  7:      $K^{\prime }\leftarrow K$  8:      **for** k=0,1,…,K−1 **do**  9:            $k^{\prime }\leftarrow K-1$10:            **for** $n\in E_{k}$ **do**11:                   $k^{\prime }\leftarrow k^{\prime }+1$12:                   Define a prospective tree $T^{\prime }$13:                   Train $T^{\prime }$ with $x_{\mathrm{train}}\left(X_{k,n},V\setminus V_{k}^{o}\right)$ and $y_{\mathrm{train}}\left(X_{k,n}\right)$14:                   Select $j_{\mathrm{sel},k^{\prime }}$, of which feature has highest feature importance in $T^{\prime }$15:                   $V_{k^{\prime }}^{o}\leftarrow V_{k}^{o}\cup \left\{j_{\mathrm{sel},k^{\prime }}\right\}$16:                   Discard $T^{\prime }$17:                   Define a new sub tree $T_{k^{\prime }}$18:                   $X_{k^{\prime }}\leftarrow X_{k,n}$19:                   Train $T_{k^{\prime }}$ with $x_{\mathrm{train}}\left(X_{k^{\prime }},V_{k^{\prime }}^{o}\right)$ and $y_{\mathrm{train}}\left(X_{k^{\prime }}\right)$20:                   Prune $T_{k^{\prime }}$ at depth of best accuracy on $x_{\mathrm{val}}\left(X_{k^{\prime }},V_{k^{\prime }}^{o}\right)$ and $y_{\mathrm{val}}\left(X_{k^{\prime }}\right)$21:                   $T_{k,n}\leftarrow T_{k^{\prime },0}$ (Graft $T_{k}^{\prime }$ to the leaf of $T_{k}$)22:                   $E_{k^{\prime }}\leftarrow L_{k^{\prime }}$23:                   $E_{k}\leftarrow E_{k}-\left\{n\right\}$24:                   $K^{\prime }\leftarrow K^{\prime }+1$25:            **end for**26:      **end for**27:      $K\leftarrow K^{\prime }$28:**end for**

In step (c) of Figure 4, steps (a) and (b) are repeated over all leaves of the parent tree, resulting in a child tree on each leaf. This is counted as one measurement step level. Once building trees in a measurement step level is complete, the child trees at the level now become each individual parent tree, and the terminal leaves ($E_{k}$) are now the leaves of the new parent trees. In the next measurement steps, steps (a), (b), and (c) are repeated, comprising a new measurement step level. This continues until the measurement step reaches a given maximum measurement step *M*.

Inference of trained ADT resembles that of LDTs. The inference starts from the root of the prior-tree. When inference reaches to the end of the tree, which is a linkage node, then request robots to measure variable $j_{\mathrm{sel},k^{\prime }}$. Once the requested variable is acquired, inference moves on to the connected child sub-tree based on the comparison of the measured value with the threshold at the node. This continues until inference reaches the terminal nodes which do not have any child trees. Although ADTs are composed of a much larger number of trees compared to LDTs, only a single tree at a measurement step level is activated when inference is performed with a single sample, and it results in a chain of trees. The batch inference process for test purposes with multiple samples is described in Algorithm 4.
**Algorithm 4** ADT testing process  1:**function** Predict($x_{\mathrm{test}}$, *k*, *m*)  2:      Run $T_{k}$ with $x_{\mathrm{test}}\left(X_{k},V_{k}^{o}\right)$  3:      **for** $n\in L_{k}$ **do**  4:              Find $k^{\prime }$ with which $T_{k^{\prime }}$ is connected to $T_{k,n}$  5:              **if** $k^{\prime }$ exists **then**  6:                      $X_{k}^{\prime }\leftarrow X_{k,n}$  7:                      Predict($x_{\mathrm{test}}$, $k^{\prime }$, m+1)  8:              **else**  9:                      $y_{\mathrm{pred}}\left(X_{k,n}\right)\leftarrow y_{k,n}$10:              **end if**11:      **end for**12:**end function**13:Predict($x_{\mathrm{test}}$, 0, 0)

## 3. Case Study

### 3.1. Simulation Environments

The proposed measurement selection method was tested in iFANnpp [28], a digital twin of a generic nuclear power plant developed specifically for simulations of robot operation in nuclear power plant environments and validation of ML applications with robots. Extending the previous work [29], iFANnpp is powered by process data produced by the Generic Pressurized Water Reactor (GPWR) Simulator developed by Western Services Corporation (WSC) [30], a high-fidelity full-scope simulator of an entire nuclear power plant system, incorporating bidirectional pipeline updates, realistic robotic simulation, and immersive 3D models implemented in Unreal Engine 5 with various interactive features.

In this research, the circulating water system (CWS) was selected as the target facility to monitor and diagnose. It consists of three pumps from a cooling water source, i.e., a lake, and three condensers, which cool down and condensate steam exhausted from turbines.

**Figure 5:**
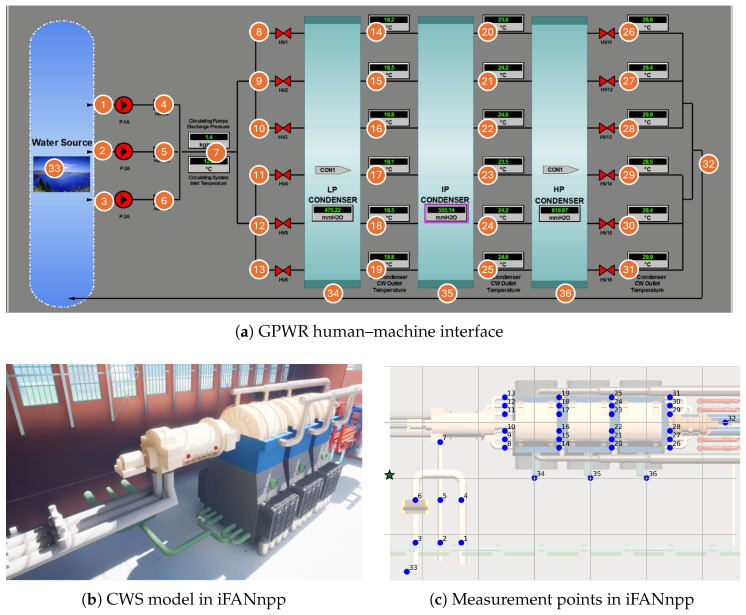
Simulation environment.

Figure 5a is the GPWR human–machine interface of the CWS. All measurement points where a robot can measure variables are plotted as round orange markers with point numbers on them. This CWS has 23 installed sensors on 19 temperature and 4 pressure variables, which are considered monitored variables in this study. It also has 70 additional measurable variables of pressure, temperature, flow rate, and pump load, of which data can be acquired at 36 physical locations. Detailed information on the variables and measurement points is listed in Appendix A.

In the iFANnpp environment, the robot simulation was performed on the lower floor of the building right below the turbine floor, as shown in Figure 5b. This floor is the space where all the major components of CWS, such as condensers, pumps, and pipes are located. 36 measurement points defined in Figure 5a are mapped on this floor as illustrated in Figure 5c with blue markers. The green star-shaped marker represents the initial position of the robot. The coordinates of the measurement points are scaled by the largest infinity norm distance between them; thus, distances are represented in a dimensionless unit. The moving speed of the robot is $16.67\times 10^{-3}\;s^{-1}$, and time required for each measurement is 2 s. To reduce the computational costs of the simulations due to the large number of instances, travel times between measurement points were individually recorded and later aggregated over the measurement sequences on each instance during post-processing of the results.

### 3.2. Simulation Conditions and Data

GPWR provides custom component-level physics model modification capabilities. In this research, leaks at different condensers were simulated by adding divergent flow paths to the air with valves in the middle of the original cooling water pipe models passing through the condensers. Thus, the acquired data was labeled in four status categories according to the status of the facility: normal, leak at HP condenser, leak at IP condenser, and leak at HP condenser. The level of leak was controlled in four levels: 0.005, 0.01, 0.02, and 0.03 which is defined by the opening degrees of the valves artificially placed to emulate the leaks from piping. It can vary from 0.0 for the fully closed state and 1.0 for the fully open state. The incident time was also varied in 15 time stamps linearly spaced by 1 min on each episode. The total number of simulation episodes was 240.

When collecting data for validation of an ML solution, ensuring diversity of data is one of the most critical aspects to consider [31,32]. In this research, all GPWR simulations were performed under varied load conditions, transitioning from 100% to 75% and back to 100% again over 17 min and 16 s with a sampling frequency of 1 Hz [33]. Water source temperature, the environmental factor that affects the performance of the nuclear power plants most significantly, was randomly sampled from a yearlong dataset from the U.S. Geological Survey database [34].

The data was normalized by min–max scaling on each feature. Gaussian noise with 0.02 standard deviation was added to all variables, and additional Gaussian noise with 0.05 standard deviation was added to measurable variables. The dataset was randomly split into three sets, training, validation, and test sets, in a ratio of 80%, 10%, and 10% in episode-wise. The validation set was used in regulating the depths of the prior-tree in GCDT models and the sub-trees in ADT. All model evaluation was performed on the test set. The resulted numbers of samples were 119,304 in the training set, 14,688 in the validation set, and 14,088 in the test set.

### 3.3. Models

To demonstrate the performance of the proposed models, LDT and ADT are tested along with the conventional MDT. When evaluating GCDT models, the prior-trees of them are evaluated separately to effectively illustrate the models’ performance along with measurement. Note that the prior-trees in LDT and ADT are identical to each other because they are a single DT trained with the same dataset, variables, and conditions.

When training and testing models, it was assumed that the facilities are in a steady state, and thus the change in data during robot operation was ignored. The depths of trees in the models were regulated to prevent overfitting. The MDT was regulated with a maximum depth of 20, where the model performance was saturated. The prior-tree of LDT and ADT were also regulated with a maximum depth of 5 because the validation accuracy started to decrease at depth 6. The sub-trees of LDT were regulated with the maximum depth of 10, which is the maximum number of measurement steps to be analyzed. ADT was regulated by the maximum number of layers of sub-treess, 10, accordingly. Nodes in all trees are regulated to have more than 10 training samples. For the classification quality criterion, Gini impurity was used.

The number of sub-trees in the trained LDT model was 32, which is equal to the number of the leaves in the prior-tree. Meanwhile, the number of sub-trees of the trained ADT model was 1095.

## 4. Results

### 4.1. Measurement Sequence Generation

**Figure 6:**
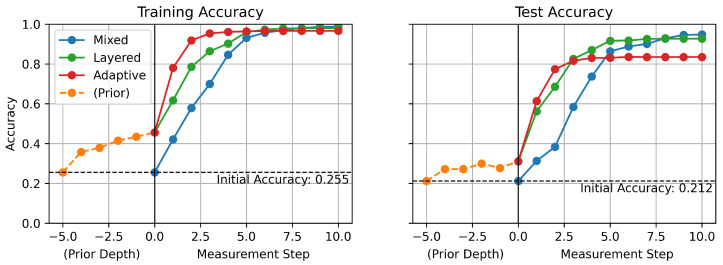
Accuracy over measurement steps.

Figure 6 shows the accuracy of the models over measurement step both on the training and test sets. The measurement step denotes in what order the robot is measuring the variable. The proposed models, LDT and ADT, outperform the conventional MDT model, especially at the early stage of measurement steps.

As stated earlier, the accuracy of the models starts from initial accuracy at measurement step 0, which is before starting measurements, and improves gradually as the measurement step increases. At step 0, MDT starts with initial accuracy, which is the random guess model performance. However, LDT and ADT start from the accuracy increased by a prior-tree with monitored values before they start at their first measurement step. Even though the accuracy increase by the prior-tree is relatively small, it creates a significant difference at the early stages of the measurement processes. The performance difference between the MDT and two GCDT models is the highest at measurement steps 2 and 3. Even though these models reach similar accuracy when the measurement step is large enough, the proposed GCDT models provide a significant advantage in achieving higher accuracy in the early stages. It also means fewer mobile measurements are needed for robots to diagnose the ongoing faults so that faster decisions can be made for appropriate responses.

It is also worth noting that ADT converges to its highest accuracy most quickly among all models, but its highest test accuracy is lower than any other model’s. Because ADT has more complex architecture, it seems to result in an overfitting issue. Therefore, while ADT could play the best model in cases when early inference is important, LDT could play a better model in cases when enough time is allowed and final accuracy is a more important factor.

**Figure 7:**
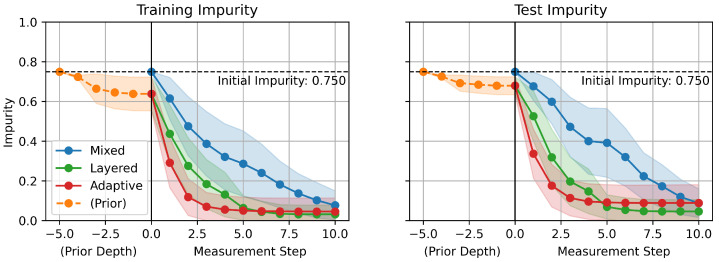
Impurity over measurement steps.

Figure 7 shows the impurity over measurement steps. The impurity of nodes in a DT $G_{n}$ is given by the statistics of the training data samples reached to the nodes [16].

(1) $$G_{n}=1-\sum_{c=1}^{C}p_{n}{\left(c\right)}^{2}$$

where C∈N is the number of classes, and $p_{n}\left(c\right)$ is the ratio of samples reached to the node *n* with the true label *c*, which satisfies $\sum_{c=1}^{C}p_{n}\left(c\right)=1$. In contrast to accuracy, which is a metric that evaluates the performance of the model over the whole dataset, impurity is a metric that can be evaluated on a single instance. This is a critical aspect of impurity as a metric because the models eventually function on a single instance, which is the current one, when deployed to a real-time online monitoring system. Therefore, impurity provides a perspective on how the model gets confident in its inference during each robot measurement scenario. Lines with markers represent mean values of impurity over the given datasets, and filled-in areas represent distribution of impurity with 0.5 standard deviation from the mean values.

Similarly to the accuracy increases with the measurement steps in Figure 6, the impurity distinctively decreases over the measurement steps, which illustrates that the models become more confident in their prediction as measurements proceed. The proposed GCDT models demonstrate a significant advantage also in decreasing uncertainty of predictions faster in comparison to the MDT.

**Figure 8:**
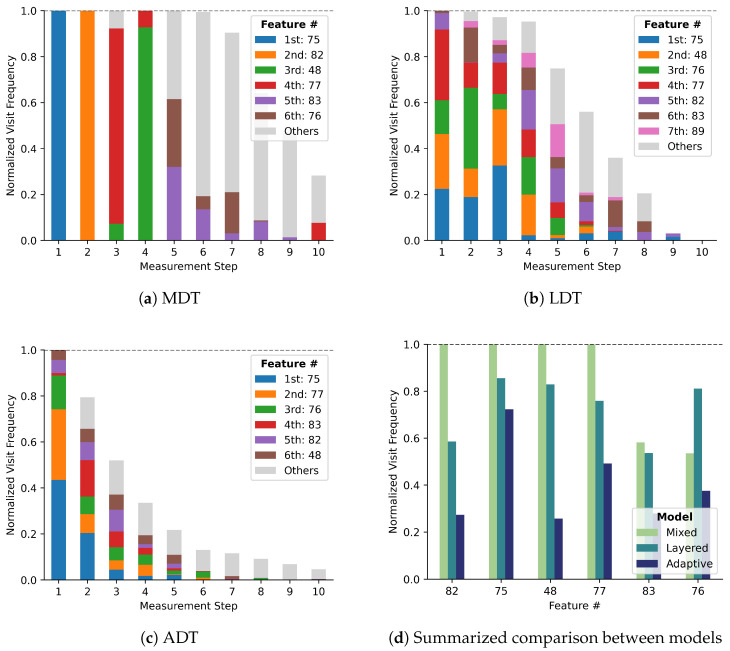
Selection frequencies of the features.

Figure 8a–c illustrates which variables are required by each model most frequently. The height of the bars represents the normalized frequency of being selected at each step, which could be up to 1.0. The six most frequently required features during inference are selected, and colors are assigned in the order of the total number of times they were selected over all measurement steps. Frequencies of all other features were aggregated and represented in the gray color bars.

In the first measurement step, feature #75 alone is always chosen regardless of any condition, because all inference starts from a single root node. However, the different features were selected at the first measurement step when using LDT and ADT. This demonstrates that the first measurement is required by multiple leaf nodes of the prior-tree in GCDT models. More varied feature selection of GCDT models continues over all other measurement steps. It implies that the proposed GCDT models more adaptively make decisions on measurement selection.

The total height of the bars at each measurement step represents the ratio of the instances that require measurements at the corresponding step number. GCDT models stop their measurement at earlier stages compared to the MDT model, because they converge to their best prediction and attain a state that does not require additional measurements.

The overall measurement frequencies of six features are summarized in Figure 8d. Four features, #82, #75, #48, and #77, are always used in inference with the MDT, which signifies that it relies on fewer variables in the target facilities. However, GCDTs do not have any feature that is used in all cases. Even the most frequently chosen feature #75, is selected with a frequency less than 1.0, which demonstrates that those are used frequently but not always. The diversity of selected features explains how adaptively measurement selection is achieved by the proposed GCDT models.

### 4.2. Performance Evaluation in Physical Space

**Figure 9:**
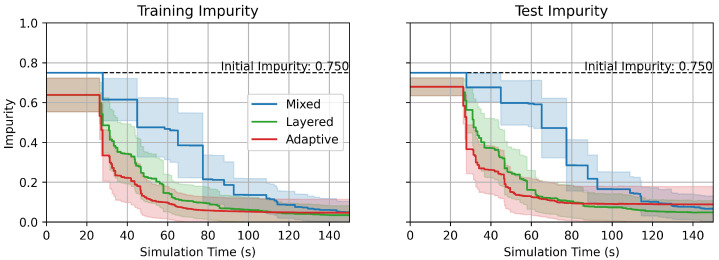
Impurity over time in robot measurement simulation in the iFANnpp.

With the measurement sequences generated from the DT models, the robot measurement simulation was performed in the iFANnpp environment to validate the efficacy of proposed models in physical spaces. The robot travel time between the measurement points was recorded, and the impurity was evaluated at each measurement point at the time of arrival. The result of the simulation is summarized in Figure 9. Note that Figure 9 is a reconstruction of Figure 7, with a different *x*-axis domain, time instead of discrete measurement steps. The *x*-coordinates of the samples in the plot were varied depending on the time stamps of the data measurements with robots. The figure also well illustrates the superiority of the GCDT models in achieving a lower impurity faster than the MDT in physical spaces, as it is in the discrete logical measurement step criterion.

**Figure 10:**
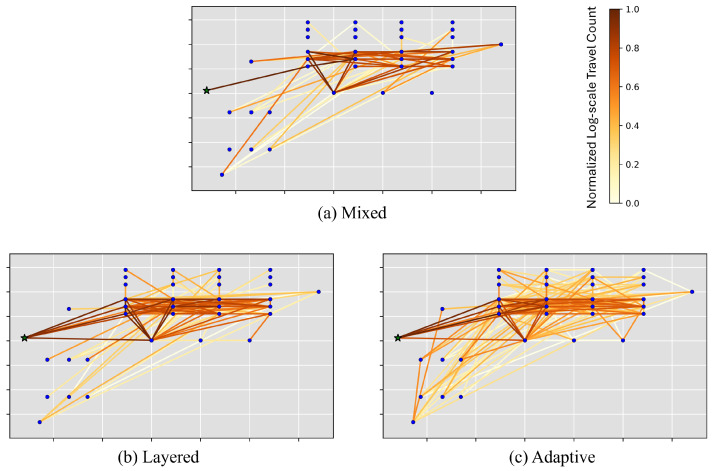
Robot paths traveling between measurement points up to step 5 in the iFANnpp environment.

Figure 10 illustrates the traveled paths between the measurement points up to measurement step 5 in iFANnpp. The color represents normalized log-scale travel counts on the paths. GCDT models result in more diverse paths traveled by the robot (Figure 10b,c) compared to the traveled paths with the MDT model (Figure 10a). It is also observed that only a single path from the robot’s initial position is traveled with MDT, while there are multiple starting scenarios for the robot with LDT and ADT. This is also another demonstration that the proposed GCDT models choose the paths more adaptively to the prior information in addition to the statistics of selected features in Figure 8.

In summary, all three DT models showed their capabilities to fulfill two objectives of the adaptive online fault diagnosis framework proposed in this research: providing next measurement selection on each step and making predictions on the status of the target facilities in each step. The proposed GCDT models, LDT and ADT, outperform the conventional MDT model in prediction with lower uncertainty in the early stages of measurement steps and require less time in achieving a certain level of uncertainty.

## 5. Conclusions

In this study, grafted GCDT models are proposed and validated, demonstrating their superior performance in measurement selection in the iFANnpp environment. The core idea of GCDT is dividing features into two categories, monitored and measurable variables, having separate sub-modules trained with limited variables, and grafting the sub-modules in an appropriate way. The architecture, having a prior-tree dedicated to initial inference with monitored variables and consequent sub-trees dedicated to further inference and measurement selection, enables the proposed models to excel in quicker diagnosis both in required number of measurements and also in required time. The proposed method was validated in a case study on CWS in a nuclear power plant using the iFANnpp digital twin and demonstrated how effectively GCDTs can achieve their goals.

As stated earlier, DT is the base model for many powerful ensemble models in a wide range of applications. Thus, it is expected that GCDT model architecture could have further impact when the core idea of the proposed models is adopted by those ensemble models. Indeed, the approaches used in the GCDT models can also be applied to other ML models as well beyond the DT-based ones. The core components of a GCDT, a prior-tree and sub-trees, do not have to be a tree model. The prior-tree can be replaced by any type of model as long as it splits training samples into a number of clusters with monitored variables. The sub-trees can also be replaced by any type of model as long as it can provide a prediction and measurement sequence and consecutive prediction with limited measurable variables. Challenges coming from the unique characteristics of those ML models should be addressed to extend the approaches of the GCDT models further. Exploration of the measurement selection problem under transient conditions, which results in different values depending on the time of measurement, is also to be addressed. It would make the proposed method step forward from the limitation of the stationary data assumption in this study as well. Extending the presented methods for multi-robot scenarios is another topic that would make further impacts. Researchers are invited to explore further improvements of GCDT models and extend their application not only to the nuclear energy sector but also to other industries, including but not limited to aerospace, renewable energy, and manufacturing, to enhance their safety and productivity.

## Data Availability

Dataset available on request from the authors.

## Appendix A. List of Variables

**Table A1:** List of variables and measurement points.

Feat #	Measurement Point #	Description	Type	Monitored
0	4	CWP-A discharge	pressure	-
1	4	CWP-A discharge	temperature	-
2	5	CWP-B discharge	pressure	-
3	5	CWP-B discharge	temperature	-
4	6	CWP-C discharge	pressure	-
5	6	CWP-C discharge	temperature	-
6	5	CWP discharge merge joint	pressure	O
7	5	CWP discharge merge joint	temperature	O
8	7	CWP discharge diverge joint	pressure	-
9	7	CWP discharge diverge joint	temperature	-
10	14	IP condenser intake A	pressure	-
11	14	IP condenser intake A	temperature	O
12	15	IP condenser intake B	pressure	-
13	15	IP condenser intake B	temperature	O
14	16	IP condenser intake C	pressure	-
15	16	IP condenser intake C	temperature	O
16	17	IP condenser intake D	pressure	-
17	17	IP condenser intake D	temperature	O
18	18	IP condenser intake E	pressure	-
19	18	IP condenser intake E	temperature	O
20	19	IP condenser intake F	pressure	-
21	19	IP condenser intake F	temperature	O
22	20	HP condenser intake A	pressure	-
23	20	HP condenser intake A	temperature	O
24	21	HP condenser intake B	pressure	-
25	21	HP condenser intake B	temperature	O
26	22	HP condenser intake C	pressure	-
27	22	HP condenser intake C	temperature	O
28	23	HP condenser intake D	pressure	-
29	23	HP condenser intake D	temperature	O
30	24	HP condenser intake E	pressure	-
31	24	HP condenser intake E	temperature	O
32	25	HP condenser intake F	pressure	-
33	25	HP condenser intake F	temperature	O
34	26	HP condenser discharge A	pressure	-
35	26	HP condenser discharge A	temperature	O
36	27	HP condenser discharge B	pressure	-
37	27	HP condenser discharge B	temperature	O
38	28	HP condenser discharge C	pressure	-
39	28	HP condenser discharge C	temperature	O
40	29	HP condenser discharge D	pressure	-
41	29	HP condenser discharge D	temperature	O
42	30	HP condenser discharge E	pressure	-
43	30	HP condenser discharge E	temperature	O
44	31	HP condenser discharge F	pressure	-
45	31	HP condenser discharge F	temperature	O
46	32	condenser discharge merge joint	pressure	-
47	32	condenser discharge merge joint	temperature	-
48	34	LP condenser	pressure	-
49	34	LP condenser	temperature	-
50	35	IP condenser	pressure	O
51	35	IP condenser	temperature	-
52	36	HP condenser	pressure	O
53	36	HP condenser	temperature	-
54	36	HP condenser	pressure	O
55	36	HP condenser	temperature	-
56	33	water source	pressure	-
57	33	water source	temperature	-
58	4	CWP-A discharge	flow	-
59	5	CWP-B discharge	flow	-
60	6	CWP-C discharge	flow	-
61	7	CWP discharge	flow	-
62	26	HP condenser discharge A	flow	-
63	27	HP condenser discharge B	flow	-
64	28	HP condenser discharge C	flow	-
65	29	HP condenser discharge D	flow	-
66	30	HP condenser discharge E	flow	-
67	31	HP condenser discharge F	flow	-
68	32	condenser discharge merge joint	flow	-
69	1	CWP-A intake	flow	-
70	1	CWP-A intake	load	-
71	2	CWP-B intake	flow	-
72	2	CWP-B intake	load	-
73	3	CWP-C intake	flow	-
74	3	CWP-C intake	load	-
75	8	LP condenser intake A	flow	-
76	9	LP condenser intake B	flow	-
77	10	LP condenser intake C	flow	-
78	11	LP condenser intake D	flow	-
79	12	LP condenser intake E	flow	-
80	13	LP condenser intake F	flow	-
81	14	IP condenser intake A	flow	-
82	15	IP condenser intake B	flow	-
83	16	IP condenser intake C	flow	-
84	17	IP condenser intake D	flow	-
85	18	IP condenser intake E	flow	-
86	19	IP condenser intake F	flow	-
87	20	HP condenser intake A	flow	-
88	21	HP condenser intake B	flow	-
89	22	HP condenser intake C	flow	-
90	23	HP condenser intake D	flow	-
91	24	HP condenser intake E	flow	-
92	25	HP condenser intake F	flow	-
